# Trigeminal Neuralgia in Advanced Head and Neck Cancer: A Complex Palliative Care Case

**DOI:** 10.7759/cureus.48393

**Published:** 2023-11-06

**Authors:** Carolina Vidal, Rosário Vidal

**Affiliations:** 1 Unidade de Cuidados Paliativos, Hospital do Divino Espírito Santo de Ponta Delgada, Ponta Delgada, PRT

**Keywords:** advanced head and neck cancer, baclofen, low-dose ketamine, secondary trigeminal neuralgia, supportive and palliative care

## Abstract

Trigeminal neuralgia (TN) is a chronic neuropathic pain disorder characterized by paroxysms of electric shock-like or stabbing pain in the face. This condition is associated with poor quality of life. First-line treatment includes carbamazepine or oxcarbazepine, but some cases show refractory symptoms to this approach. We describe a challenging case of secondary TN due to an advanced head and neck cancer managed by a palliative care team.

## Introduction

Trigeminal neuralgia (TN) is a debilitating neuropathic pain condition with a major impact on physical, psychological, and social well-being [1]. Patients facing an advanced head and neck cancer experience high burdens of physical and psychosocial suffering. In this article, we describe the challenging case of a patient with advanced head and neck cancer with metastasis to the left temporal fossa, cavernous sinus, and perineural invasion of the third trigeminal root, leading to severe facial pain that was unresponsive to multiple treatment approaches.

## Case presentation

A 58-year-old male, with a history of alcoholism and smoking, was diagnosed with squamous cell carcinoma of the gingival margin of the third oral quadrant. The cancer extended between the symphysis and the left angle of the mandible, with bone invasion. He was initially submitted to a complex and mutilating surgery of segmental pelviglossomandibulectomy, cervical lymph node dissection, and reconstruction with osteomyocutaneous flap of the left fibula on February 2020. He was then treated with chemoradiotherapy with carboplatin. However, by May 2021, the disease progressed to the left temporal fossa and cavernous sinus.

**Figure 1:**
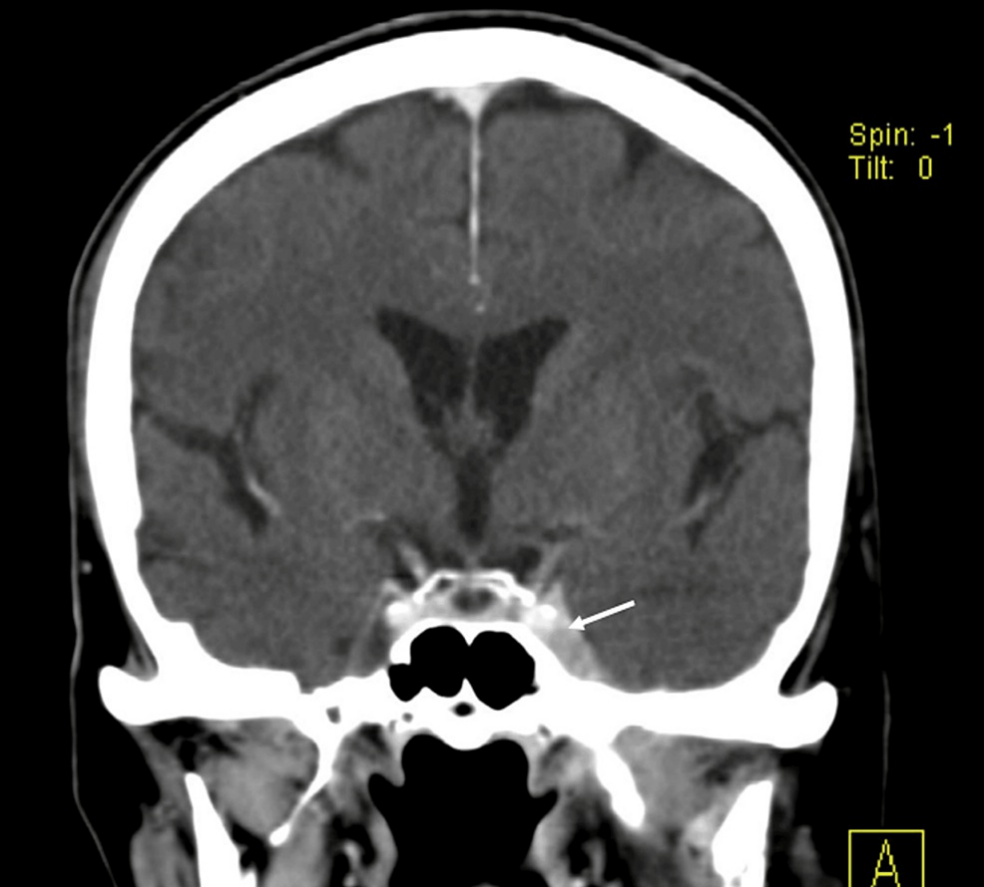
Brain computed tomography (coronal plane) showing tumor involvement of the left cavernous sinus

At this point, a new chemotherapy regimen with carboplatin and paclitaxel was initiated. However, despite this new chemotherapy regimen, the computed tomography scan revealed disease progression to the foramen oval with invasion of the third trigeminal root (Figure 1).

The patient was admitted to our Palliative Care Unit on January 12, 2022 with severe left facial pain reaching a peak intensity of 8 points on the numeric pain scale. The patient described continuous pain with intense attacks located in the maxillary and mandibular trigeminal branches. Pain was triggered by light touch and activities such as shaving. Regarding functionality, the patient walked without assistance, performed self-care, and was fully alert with a score of 80% on the Palliative Performance Scale [2]. The patient could only feed himself through a syringe or straw, due to oral deformities caused by the surgery. He also showed high social vulnerability, as he did not maintain significant relationships with relatives and lived with an elderly friend.

Despite being treated with carbamazepine 800 mg/day, duloxetine 60 mg/day, dexamethasone 4 mg/day, paracetamol 3 g/day, and fentanyl 150 mcg/h via transdermal patch, the patient had severe attacks of facial pain, requiring six oral morphine rescues per day (36 mg each). The patient had already been treated with pregabalin up to 300 mg/day with little response, which had been replaced by carbamazepine in previous contacts with our team.

Our palliative care team assumed a mixed etiology for the patient's pain, which included nociceptive pain due to facial bone and soft tissue involvement and neuropathic pain due to trigeminal nerve invasion.

During the patient's stay in our unit, we gradually increased the carbamazepine doses up to 1200 mg/day, but after 10 days, the patient developed symptoms of neurotoxicity such as nausea, vomiting, dizziness, and severe ataxia. At this point, serum carbamazepine concentrations were supratherapeutic and the carbamazepine dose was reduced to 600 mg/day. Serum sodium and liver function remained within the normal ranges. Carbamazepine levels then dropped into the therapeutic range, toxicity was solved, but the patient remained without pain control.

The team consulted with the Neurology department and treatment with oxcarbazepine was not undertaken due to the severity of the cerebellar toxicity and lack of pain control with carbamazepine. Recognizing the nociceptive component of pain, we concurrently increased the fentanyl dose to 250 mcg/h, via a transdermal patch.

Opioid rotation for subcutaneous morphine was attempted but the patient developed frequent signs of subcutaneous malabsorption. Additionally, the patient had fragile venous access, so we decided to return to a transdermal fentanyl prescription.

Nevertheless, the patient continued to experience severe pain and required multiple subcutaneous morphine rescues per day. Botulinum toxin injections, using the “follow the pain” technique, were performed by an experienced neurologist, but unfortunately there was no improvement in pain control.

Given the high doses of opioids achieved, we initiated ketamine treatment to reduce opioid hyperalgesia and rescue analgesia via NMDA antagonism. The patient started an oral solution of ketamine at an initial dose of 40 mg/day. Previous treatment with olanzapine was maintained to prevent neuropsychiatric effects, plus rescue doses of midazolam. Despite these preventive measures, the patient developed intense agitation, hallucinations, and sialorrhea, both refractory to midazolam and atropine. Ketamine was then discontinued with no long-term benefit.

Given the patient's refractoriness to high doses of morphine and fentanyl, treatment with methadone was considered, but unfortunately, access to this drug remains very difficult in our country. Then, we explored another therapeutic approach using baclofen. Remarkably, the patient exhibited a good analgesic response with baclofen 60 mg/day as adjuvant therapy. 

In addition to pharmacological interventions, recognizing the significant impact of psychosocial pain, the patient received crucial psychological and social support, as well as daily treatment from a physiotherapist, speech therapist, and occupational therapist.

After a 47-day stay in our Palliative Care Unit, the patient was discharged with excellent pain control, undergoing treatment with fentanyl 250 mcg/h transdermal patch, carbamazepine 600 mg/day, duloxetine 60 mg/day, and baclofen 60 mg/day, requiring less than three oral morphine rescues (50 mg each) per day.

At discharge, the patient was autonomous in activities of daily living and resumed cancer treatment with nivolumab. At home, the patient kept symptoms under control and was able to endure arduous tasks such as agricultural activities. He was followed up by our community palliative care team, showing adequate symptom control for almost 10 months. However, in December 2022, there was a clear progression of the disease and a malignant wound with bone fragments developed on his left mandibular area. The patient was then readmitted and died on December 13.

## Discussion

The diagnosis of TN requires recurrent paroxysms of unilateral facial pain restricted to the trigeminal distribution, lasting seconds to two minutes. The pain is characterized by its severity, often described as electric shock-like shooting, stabbing, or sharp quality. It is also typically precipitated by innocuous stimuli [3]. Although paroxysmal facial pain is the hallmark of TN, 24 to 49% of patients report continuous or long-lasting pain between paroxysmal attacks [4].

Three types of TN have been identified, namely classical, secondary, and idiopathic TN [1]. The classic type, accounting for 80 to 90 percent of the cases, is caused by intracranial vascular compression of the trigeminal root [4]. Less frequent etiologies of TN include multiple sclerosis and tumors, most frequently meningioma and squamous cell carcinoma.

Regarding TN treatment, antiepileptic drugs such as carbamazepine and oxcarbazepine are the first-choice drugs for long-term treatment [1,4]. Adverse effects of carbamazepine include drowsiness, nausea, vomiting, ataxia, rash, hyponatremia, liver damage, and agranulocytosis [1]. Slow titration may minimize, but not prevent, these effects [1,4]. Oxcarbazepine is also an effective drug for TN. Adverse effects of oxcarbazepine are similar to carbamazepine, including nausea, vomiting, hyponatremia, rash, and drowsiness. However, some experts assume better tolerability of oxcarbazepine compared to carbamazepine [4]. 

Despite the compressive etiology of the patient's pain, we did not increase the dose of dexamethasone, since this would compromise immunotherapy treatment with Nivolumab.

Other options, with lower evidence, such as botulinum toxin type A, baclofen, lamotrigine, and phenytoin may be used either alone or as add-on therapy when first-line drugs fail due to efficacy or tolerability [1,5]. In this case, we used botulinum toxin with scarce benefit. Despite the lower evidence for baclofen [5], in this case, baclofen used as add-on therapy enabled optimal pain control with no adverse effects.

In classical TN, decompressive surgery has an important role. However, in this case of secondary TN due to direct nerve invasion, in the context of advanced disease, this option was not advisable. Eventually, if pain remained refractory, percutaneous radiofrequency treatment of the Gasserian ganglion would be a reasonable alternative.

## Conclusions

We describe a complex case of secondary TN due to advanced head and neck cancer. The patient showed refractory pain to carbamazepine and experienced toxicity despite slow titration of this drug. He was unresponsive to high doses of opioids and classic adjunct therapy. Botulinum toxin failed to provide the expected analgesia. However, the introduction of baclofen, a less well-established option for TN, enabled optimal pain control in this patient. 

Although baclofen is not an uncommon choice for the treatment of TN, in this case, given the multiple concurrent etiologies of the physical pain and the high burden of psychosocial suffering, the team did not anticipate such a remarkable improvement in pain control with baclofen. We also emphasize the importance of an interdisciplinary approach while caring for head and neck cancer patients.
